# Plan That Safeguards Healthy Labour

**Published:** 1920-02-07

**Authors:** 


THE PUBLIC WELL-BEING—(*continued*).

### PLAN THAT SAFEGUARDS HEALTHY LABOUR.

The question of the subsequent career of consumptives who have come under the control of the Tuberculosis Officer is dealt with by Dr. Sidney Barwise in his annual report as medical officer of health for Derbyshire. With regard to farm-colony treatment, he points out that the farm colony does not mean that the tuberculous man or woman is to be engaged on farm work, which is skilled and laborious and necessitates a seven-days' week. By a farm colony is meant the housing of consumptives in detached houses, where forestry, nursery gardening, horticulture, fowl-keeping, pig-keeping, bee-keeping, woodwork, and similar handicrafts can be carried out—the products of the labour being sold through one organisation. A better term would be a "craft colony."

It will be necessary, he says, to come to terms with the labour organisations for certain industries to be earmarked as suitable for the consumptive to be engaged in, so that there shall be a good market for the products of his subsidised labour, and that the consumptive shall not be in competition with healthy labour. They must think out where the process of segregating consumptives is going to end, and to get a true perspective of the problem they must remember that the great bulk of the population is suffering or has suffered from tuberculosis.

The development of "craft colonies" will, it is hoped, meet the case of those patients whose occupations are obviously unsuitable, or who are without occupation.

It may be taken as axiomatic that no individual who has suffered from established pulmonary tuberculosis ever regains his full working capacity. Parenthetically it may be observed that occupations involving severe prolonged exertion, even if completely out-of-doors, are quite unsuitable for the post-tuberculous. It would be well if this loss of working capacity, and, therefore, of earning power, were generally recognised. A post-tuberculous subject may be capable of efficient and of brilliant and original work, especially when such work is of a skilled and technical nature; but in heavy manual work the consumptive cannot with impunity attempt to compete with the healthy. It is only just, therefore, that tuberculous subjects should be prepared to accept a lower wage, working shorter hours than the healthy, otherwise it will be impossible to find them employment.

This point has a definite bearing on the "minimum wage," now probably a permanent feature of industry, and he commends the question to the sympathetic consideration of the various trade unions and other industrial organisations, and makes the suggestion that Tuberculous Officers should certify the "percentage efficiency" of the consumptive, and that the various trade unions should honour such certificates.

Another difficulty which must be stated and faced is that most of the friendly societies stop sickness benefit as soon as the consumptive can do any work. It may be good for the patient to do half a day's work, but he cannot live, let alone build up his "life capital," on half rations. Here, again, special certificates issued by the Tuberculous Officer for a definite period should permit the patient to continue to receive sickness benefit while doing a certain amount of work under medical advice as part of his treatment.

As regards "craft colonies," they must be limited (*a*) to advanced "open" cases, still able to work, who may be a danger to others, and (*b*) to cases that have been treated in sanatoria and require an additional stay of twelve months or so in the open air to effect a complete arrest of the disease.

Dealing generally with the subject, Dr. Barwise declares that the only chance of "stamping out" the disease would appear to be the discovery of some form of vaccination against tuberculosis.

